# Remote Sensing Greenness and Urbanization in Ecohydrological Model Analysis: Asia and Australasia (1982–2015)

**DOI:** 10.3390/s19214693

**Published:** 2019-10-29

**Authors:** Danlu Cai, Klaus Fraedrich, Yanning Guan, Shan Guo, Chunyan Zhang, Rui Sun, Zhixiang Wu

**Affiliations:** 1 Danzhou Investigation & Experiment Station of Tropical Crops, Ministry of Agriculture and Rural Affairs, Danzhou 571737, China; wzxrri@163.com; 2 Institute of Remote Sensing and Digital Earth, Chinese Academy of Sciences, Beijing 100101, China; guan@irsa.ac.cn (Y.G.); guoshan@irsa.ac.cn (S.G.); zhangcy@radi.ac.cn (C.Z.); 3 Max-Planck-Institute for Meteorology, 20146 Hamburg, Germany; 4 Rubber Research Institute, Chinese Academy of Tropical Agriculture Sciences, Haikou 571101, China

**Keywords:** ecohydrological models, climate and anthropogenic induced changes, vegetation greenness, dry gets drier and wet gets wetter

## Abstract

Linking remote sensing information and ecohydrological models to improve understanding of terrestrial biosphere responses to climate and land use change has become the subject of increased interest due to the impacts of current global changes and the effect on the sustainability of human lifestyles. An application to Asia and Australasia (1982–2015) is presented, revealing the following results: (i) The broad distribution of regions with the enhanced vegetation greenness only follows the general pattern as for the whole, without obvious dependence on regional or climate fluxes ratios. That indicates a prevailing increasing greenness over land due to both the impacts of current global changes and the sustainability of human lifestyles; (ii) regions with vegetation greenness reduction reveal a unique distribution, concentrating in the water-limited domain due to the impacts of external (climatically “dry gets drier and wet gets wetter”) and internal (anthropogenically increased evaporation) changes; (iii) the external changes of dryness diverge at the boundary separating energy from water-limited regimes, and the internal changes indicate large-scale afforestation and deforestation) that occur mainly in China and Russia due to a conservation program and illegal logging, respectively, and a massive conversion of tropical forest to industrial tree plantations in Southeast Asia, leading to an increased evaporation.

## 1. Introduction

Changes in vegetation greenness have been reported at regional and continental scales on the basis of forest inventory and satellite measurements [1,2,3,4]. Long-term changes in vegetation greenness are driven by multiple interacting direct factors (human land-use management) and indirect factors (such as climate change, CO_2_ fertilization, nitrogen deposition, and recovery from natural disturbances) [5,6,7,8,9,10].

Recent satellite data reveal a greening pattern induced by direct factors (human land use), which enhances the vegetation greenness as it is strikingly prominent in China and India, notwithstanding the impact of indirect factors [11]. An enhanced vegetation growth has broad implications for surface energy, water and carbon budgets, and ecosystem services across multiple scales [7,12,13,14]. Linking remote sensing and climate and ecosystem models to provide improved understanding of terrestrial biosphere responses to climate and land use change has become the subject of increased interest due to the impacts of current global changes and sustainability of human lifestyles [2,11,15].

The reliable detection and attribution of changes in vegetation growth are prerequisites for the development of strategies for the sustainable management of ecosystems [3]. However, human exploitation of land will remain a complex dynamic endeavour, and the underlying mechanisms are not yet fully understood [5,11]. To quantify impacts of climate change and anthropogenic activities on land surface dynamics, an ecohydrological modelling approach has been introduced [16,17], which is based on two nondimensional flux ratios as ecohydrological variables. They separate energy and water supply to distinguish climate from land use-induced change effects.

The application of the ecohydrological conceptual model has been proved and expanded to regional scales by combining remote sensing vegetation information and reanalysis data in an ecohydrological state space, spanned by relative excess energy *U* and excess water *W* [18,19]. Here, the diagnostic is used, combined with nighttime lighted city distribution (urban) to answer the following questions: Do the increased/decreased regions of greenness show a general pattern? Are there any differences between the whole study regions, significant ecohydrologically changed regions (significant *U,W* changes), and urban areas? How do climate changes and anthropogenic activities contribute to those regions? The aim of the following analysis is to attribute and quantify these changes to climatic or human-induced causes across Asia and Australasia. Our evaluation comprises ecohydrological surface information of the changing rainfall–runoff chain jointly with remote sensing observations of vegetation and urbanization, which, to our knowledge, has not been performed.

## 2. Data and Method

Data on climate, vegetation greenness, and urbanization were provided by ERA-Interim, GIMMS NDVI (1982–2015 averages, see [20,21]) and DMSP/OLS nighttime light (2012, see [22,23]). The spatial resolutions of the ERA-Interim and GIMMS vegetation greenness were 0.75° by 0.75° and 8 km by 8 km, respectively. The global DMSP/OLS nighttime stable light from NOAA National Geophysical Data Center provided the information for urbanization, given the brightness range 0 < *DN* < 63 and using the spatial resolution of about 1 km or 30 arc-seconds. The spatial resolutions were resampled according to the relatively coarse resolution of ERA-Interim into 0.75° by 0.75° by bilinear resampling.

### 2.1. Data Preprocessing

Unlike traditional sociodemographic research using administrative boundaries, population, size or density, and economic indicators to define a city, remote sensing techniques are employed in this study to analyze continuous spatial variation in nighttime light intensity of development or degree of modification. To reduce the effects of overglow [22,24], only spatially contiguous lighted pixel units (subscript “*i*”) with *DN_i_* ≥ 12 are identified as cities (for detailed descriptions of data quality control and threshold selection, see Small et al. [24]). The area of each pixel within spatially contiguous lighted areas, *a(j)*, is defined as *a_i_*, where *j* corresponds to the sequence of spatially contiguous areas exceeding the brightness threshold, and *i* corresponds to the number of pixels in each of these areas. Thus, a city (numbered by “*j*”) size is characterized by size (*j*) *=* $\sum_{i}a_{i}\left(j\right)$.

### 2.2. Ecohydrological State Space Analysis

Ecohydrological analysis is a physical approach to diagnose the controlling factors of the rainfall–runoff chain on watershed scale [16,17]. Water and energy are supplied by precipitation *P* and net radiation *N* or potential evapotranspiration. In a climatological mean, these fluxes are balanced by the partitioning of evapotranspiration *E* and runoff *Ro* and by evapotranspiration *E* and sensible heat flux *H*, respectively. The energy flux units are in water flux equivalents, m yr^−1^.

*P = E + Ro or  1 = E/P + Ro/P* (1)

*N = E + H or  1 = E/N + H/N* (2)

Energy and water balance equations are supplemented by an empirical equation of state, which, in the framework of Budyko (1974 [25]), characterizes the rainfall–runoff chain. Here, Schreiber’s equation ([26]; for a stochastic interpretation, see [27]) was used.

*Ro = P exp(−N/P)* (3)

For ecohydrological analysis, water and energy fluxes are separated by introducing relative excess energy *U* and relative excess water *W*. These flux ratios characterize energy and water fluxes, which are unused by the ecosystem (and, therefore, in excess); that is, excess energy is available for atmospheric heating, and excess water is available for geomorphological formation [28].

*U = H/N = 1 − E/N* (4)

*W = Ro/P = 1 − E/P* (5)

*U = 1 + (1 − W)/ln(W)* (6)

The flux ratio of the net radiation and precipitation yields Budyko [25] dryness index:

*D = N/P = (1 − U)/(1 − W)* (7)

**Figure 1:**
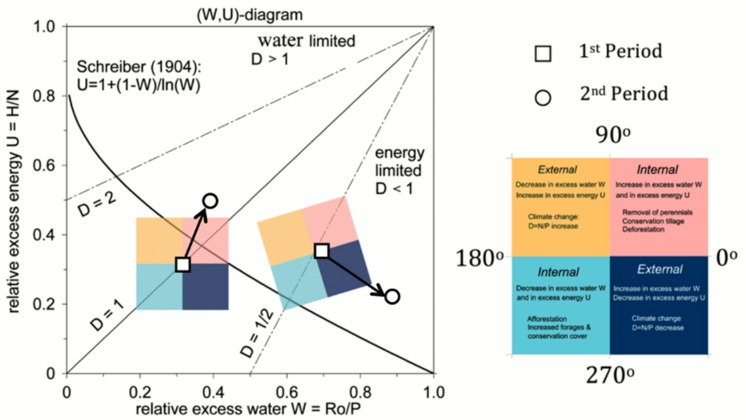
Ecohydrological states and their changes in (*U,W*) space with coordinates of relative water excess or runoff vs. precipitation and relative energy excess or sensible heat flux vs. net radiation. Lines of constant dryness or net radiation vs. precipitation and the graph of an ideal rainfall–runoff chain, *U* = 1 + (1 − *W*)/log(*W*) (see [29,30]). Squares and circles represent the ecohydrological reference, states (squares) representing the first period followed by subsequent second period (circles). Directions and lengths of the vectors (arrows) connecting first and second period determine quality and magnitudes of the causes of change (see text), as described by squares of different colors.

Surface climates are conveniently projected into the (*U,W*) space as an ecohydrological state space spanned by flux ratios to separate energy from water flux-related excesses (Figure 1). The dryness index *D* is displayed in the (*U,W*) space, separating energy (0 < *D* < 1) from water (*D* > 1) limited regimes. In addition, biomes can be suitably identified ranging from tundra (*D* < 0.3) via forests (0.3 < *D* < 1) and steppe/savanna (1 < *D* < 2) to semidesert (2 < *D* < 3) and desert (*D* > 3) [15,25].

In the (*U,W*) space, ecohydrological changes are visualized as pieces of trajectories (or vectors, see Figure 1) whose origin and end characterize the (*U,W*) space representing the first and the subsequent second averaging period. The causes of change can be attributed to a forcing induced by contributions from external or climate and from internal or anthropogenic origin [16,17,18]:

(1) External (climate-induced) processes change the climate forcing, which can be visualized as the vector components in the direction of the negative diagonal towards the second yellow or the fourth dark blue quadrant. For example, given *E* = constant, for increasing (decreasing) aridity d*D* > 0 (< 0), one obtains an increasing (decreasing) energy excess *U* = 1 − *E/N* and decreasing (increasing) water excess, *W* = 1 − *E/P*;
(2) Internal (human-induced) processes change the partitioning of the fluxes balancing the forcing, which are represented by vector components in the direction of the positive diagonal towards the first pink or the third light blue quadrant. For example, given a constant climate forcing of *P* and *N*, but changing evapotranspiration, then d*U* = −d*E*/*N* and d*W* = −d*E*/*P* represent an internal change in flux partitioning (say d*E*) affecting the watershed, such as a change in vegetation or land surface;
(3) In (*U,W*) space, the dryness ration *D* represents lines that, ending at (*U,W*) = (1,1), show slopes with magnitudes given by the inverse aridity ratio (Equation (7)). The (inverse) initial slope at (*U_1_,W_1_*) point (corresponding to the *D* = 1 − line) defines the attribution coordinates. Vector components of ecohydrological states changing from a first to a second period, which are aligned along *D*-lines (perpendicular to the *D*-lines), are attributed to internal anthropogenic control (external climate forcing). Related orthogonalization provides quantitative measures of the external and internal causes of change. In this sense, the attribution of the causes of change is quantified by the lengths of the vector components projected onto (perpendicular to) the dryness or *D* = 1 − line ending at (*U_1_,W_1_*) (for detailed calculation, see [18]).

## 3. Application of Ecohydrological Analysis: Asia and Australasia

Changes of the surface energy and water fluxes, which represent the changing rainfall–runoff chain, are evaluated in the following by employing the attribution analysis to classify and quantify the causes of change observed in Asia and Australasia. Depending on vegetation greenness increase or decrease, two domains were selected for comparative analysis: (i) The continental domain covering the administrative region of Asia and Australasia with significant ecohydrological (*U,W*) change, and (ii) the embedded smaller scale regions affected by prominent anthropogenic activity (that is, cities or urbanized areas). The periods of 1982–1998 and 1999–2015 are suggested by the climate warming tendency showing high and low values during the first and second periods, respectively [30,31].

### 3.1. Land Surface States in (U,W) Space: Means and Changes

**Figure 2:**
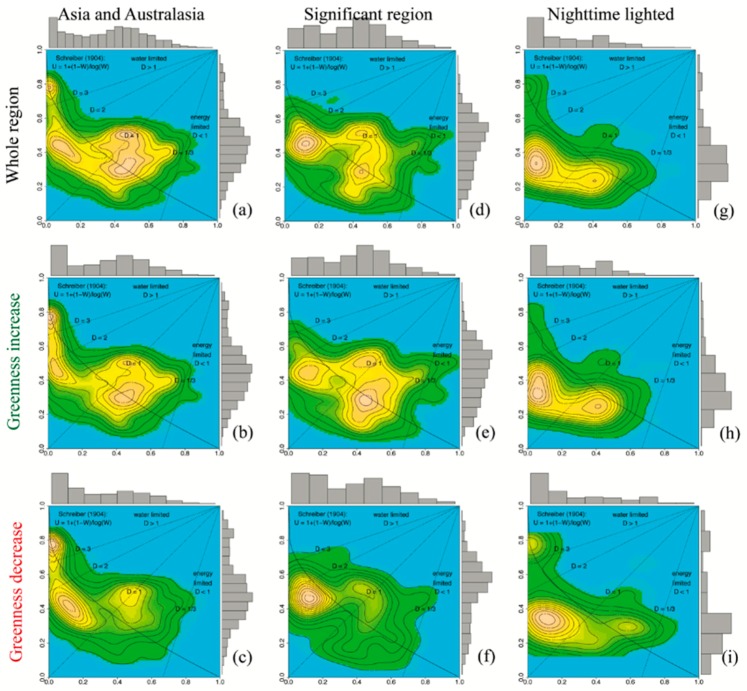
Frequency distributions of 34 year climate means in (*U,W*) state space spanned by relative excess energy (sensible heat flux vs. net radiation) and excess water (runoff vs. precipitation) in Asia and Australasia: (**a**–**c**) (*U,W*) climate means, (**d**–**f**) significant (*U,W*) change exceeding the standard deviations std(*U*) or std(*W*) from the climate means, and (**g**–**i**) (*U,W*) climate means of urbanized land surface only (that is, spatially contiguous lighted areas with *DN* ≥ 12 being classified as urbanized or cities; for detailed descriptions of data quality control and threshold selection, see text). The land surface states (upper row) are subdivided into the two categories of vegetation greenness increase or decrease from the first to the second period (middle and bottom row, 1982–1998 versus 1999–2015).

The climate mean state is presented in (*U,W*) space comprising the geographical distribution of relative excess energy *U* and excess water *W* in terms of frequencies of pixels, each of which is associated with a (*U,W*) pair. Thus, the physical states in the (*U,W*) space were characterized by a number (density) of area units, associated with long-term (*U,W*) climate means (1982–2015) obtained from the ERA-Interim dataset: (1) Administrative Asia and Australasia count 9782 pixels on the ERA-interim scale (Figure 2a,c); (2) regions of significant (*U,W*) change exceeded a standard deviation, std(*U*) or std(*W*), from the 34 year annual mean, covering about 17% (1661/9782) of Asia and Australasia (Figure 2d,f); and (3) spatially contiguous lighted cities (*DN_i_* ≥ 12) covered about 3.3% (325/9782) of Asia and Australasia (Figure 2g,i). These three states (Figure 2, upper row) were separated into two categories of increasing and decreasing vegetation greenness change (Figure 2, middle and bottom row, 1982–1998 versus 1998–2015).

The frequency distributions in (*U,W*) space characterized the climatological setting (1982–2015) as follows:

(i) Three land surface modes (Figure 2, upper row): Asia and Australasia were characterized by a trimodal distribution with peaks stretching from 1/3 < *D* < 5. Besides the peak in the very dry region (*D* > 3), the mass of Asia and Australasia was concentrated in the dryness range 1/3 < *D* < 2, which crossed the boundary separating energy from water-limited regimes. A similar distribution is found in regions of significant (*U,W*) change with minor differences on the separation line at *D* ~ 1. Nighttime lighted cities show an obvious bimodal distribution occupying wet and dry regimes (1/3 < *D* < 1 and more in 1 < *D* < 2). Compared with the regions of significant (*U,W*) change, nighttime lighted cities tended towards the origin (*U* = 0, *W* = 0) sliding away from the Schreiber curve (Equation (6)), which represents balanced states.
(ii) Vegetation increase (Figure 2, middle row): The frequency distributions of the three land surface states associated with regions where vegetation greenness increased in the second period followed a general pattern as the whole regions (Figure 2, upper row) showed no obvious dependence on regional or climate fluxes. That is in agreement with previous research, which had shown a prevailing vegetation growth over the Earth’s lands since the early 1980s when satellite data became available [8,11,29,32,33]. This indicates that regions of increasing vegetation greenness were affected by similar positive climate or human impact on greenness.
(iii) Vegetation decrease (Figure 2, bottom row): Unlike regions of increased vegetation greenness (Figure 2, middle row), climate change and anthropogenic activity contributed negatively to the three land surface modes associated with vegetation greenness reduction, and they revealed unique distribution patterns: Asia and Australasia were characterized by a trimodal distribution with one mode aligned along *D* = 1 and the other two modes separated by dryness differentiation (1 < *D* < 2 and *D* > 3). The dominant mode of Asian and Australasian greenness decrease was concentrated in the water-limited domain, and a similar distribution could be found in the nighttime lighted cities. That is, cities located in the water-limited domain were more likely to show decreasing vegetation greenness. The mass of significant (*U,W*) change was concentrated in the water-limited domain, but tended to move closer to the Schreiber (Equation (6)) curve.

In general, the pixel distributions of the three climate mean states in (*U,W*) space (Figure 2, upper row) showed structurally similar patterns without considering vegetation greenness change and for increased greenness regions (Figure 2, middle row). However, decreasing vegetation greenness regions (Figure 2, bottom row) showed different energy–water dependence and urbanization dependence as noted in the above. Thus, it is reasonable to attribute state change trajectories in (*U,W*) space to external (or climate) and internal (or anthropogenic) causes over the decreasing vegetation greenness regions and diagnose the underlying negative climate vegetation and urbanization relations.

### 3.2. Attribution of Change: Regions of Decreased Greenness

**Figure 3:**
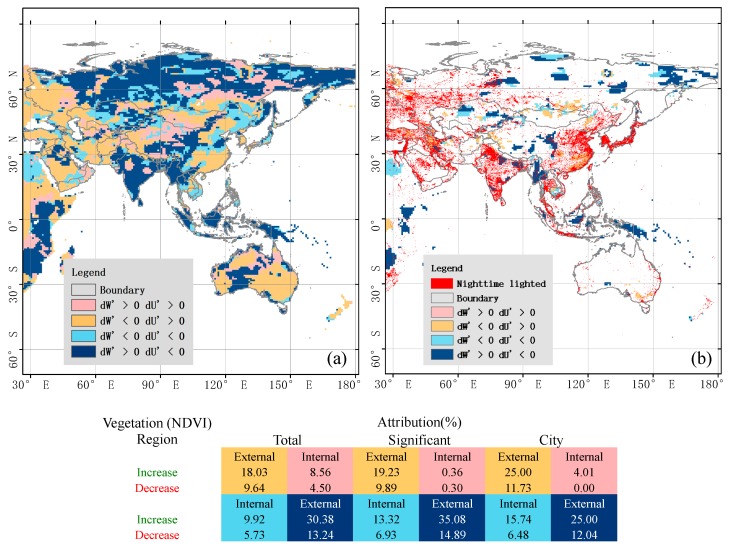
Distributions and statistics of attribution classes of (**a**) ecohydrological and (**b**) significant ecohydrological change in Asia and Australasia separating internal from external causes in three land surface states, characterizing the mean and its change and the mean of urbanization of the land surface.

**Figure 4:**
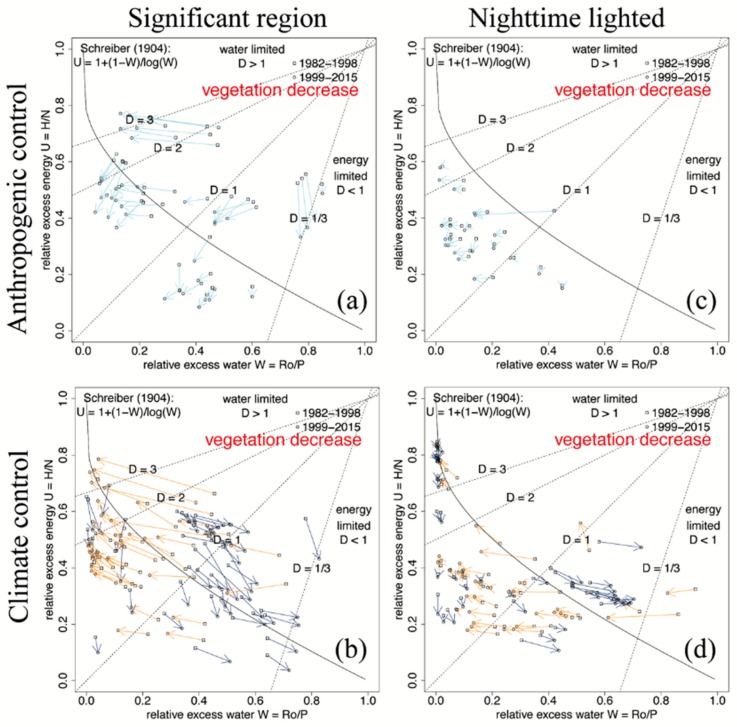
Trajectories of excess energy and water (*U,W*) change in regions of decreasing vegetation. Directions and lengths of arrows connecting first with second period provide the attribution of change of the internal/anthropogenic and external/climate partitioning (see text): (**a**,**b**) Internal-external partitioning in regions with significant (*U,W*) change (exceeding the std(*U*) or std(*W*)); and (**c**,**d**) internal-external partitioning in urbanization (nighttime lighted) areas of Asia and Australasia.

Regions of greenness change associated with three land surface states, (i) administrative climate mean, (ii) ecohydrological significant change, and (iii) nighttime lighted urbanization, being attributed to external and internal causes are partitioned into four quadrants and first displayed geographically and statistically (Figure 3). Then, changes in (*U,W*) space are represented by pieces of trajectories to highlight and visualize vegetation greenness decreasing from the first period to the second period (Figure 4).

Attribution of external and internal causes (Figure 3): Changes in the three land surface states show both a similar percentage of area cover affected by increased versus decreased vegetation greenness (around 70% versus 30%) and regions predominantly affected by external processes (between 70% and 80%). Of the external controlling regions, the fourth dark blue quadrant and second orange quadrant represent decreasing and increasing aridity associated with increasing and decreasing W and decreasing and increasing U, respectively. Thus, geographically, most regions of Russia, from Southwest China to India, the islands in Southeast Asia, and Western Australia indicate a decreasing aridity.

Of the internally affected regions, large-scale afforestation (third light blue quadrant) occurs mainly in China, which is in agreement with previous research about the largest net gain of forest [11,34]. Deforestation (first pink quadrant) could be detected by the ecohydrological model in Russia where illegal logging is a serious issue, especially in remote areas. The extent of illegal logging is estimated to affect between 5 and 30 percent of the boreal forest [34,35]. For Southeast Asia, with a massive conversion of tropical forest to industrial tree plantations [36], this attribution analysis identifies few pixels in pink as deforestation (but more in light blue as afforestation). The possible reason is that industrial tree plantations evaporate more than tropical forests (see also Röll et al. [37]).

Trajectories of changes (Figure 4): The causes of change can be separated into anthropogenic and climate contributions (upper and lower rows, respectively). For reduced vegetation greenness, the trajectories, which visualize the significant change from the first period to the second period in (*U,W*) space, reveal the following results:

(i) Anthropogenic (internal) induced changes: Regions of significant (*U,W*) change (Figure 4a) and spatially contiguous lighted cities (Figure 4c) in Southeast Asia (Figure 3) were subjected to change in forestation (light blue arrows). Conversions of (tropical) forests to industrial tree plantations and the greening of cities led to increasing evaporation (d*E* > 0). Thus, discarding climate control (*N* and *P* constant), the anthropogenic or internal change of the flux partitioning is d*U* = −d*E*/*N* and d*W* = −d*E*/*P*. Thus, if evaporation increases (d*E* > 0), both d*U* and d*W* decrease, which leads to the light blue trajectories (Figure 4) and the light blue quadrants (Figure 3), which both characterize the conversion of forest to industrial tree plantations and the greening of cities;
(ii) Climate (external) induced changes: The regions of significant (*U,W*) change revealed a general pattern of dry remaining dry or getting dryer and wet remaining wet or getting wetter, which is accompanied with a dryness change by crossing the (*D* = 1) threshold of energy to water-limited regimes. Associated with the changing density distribution in Figure 2f, it appears that the peak in the water-limited region is enhanced, and the less obvious peak aligned along *D* = 1 will move towards the energy-limited region. Unlike regions with significant (*U,W*) changes, the spatially contiguous lighted cities did not show obvious general patterns.

Attribution analysis employed for the *30%* of the area of Asia and Australasia where vegetation greenness has changed provided the following results for decreasing vegetation: (i) Significant anthropogenic or internally induced land surface changes (light blue arrows, Figure 4a) were caused by large-scale afforestation and deforestation mainly in China and Russia supported by a conservation program and illegal logging, respectively, while the tropical Southeast Asia was subjected to a massive conversion of forestation of the industrial tree plantation. All of these changes were associated with increasing evaporation; (ii) climate-controlled or externally changed (Figure 4b) areas show that the yellow and dark blue arrows were tending to regimes of enhanced dryness or wetness, respectively. Furthermore, an excess water ration of *W* = 1 − *E/P* − 0.4 may be identified as a critical threshold from which the direction of climate change diverges, tending towards drier/wetter regimes (characterized by larger/smaller dryness *D* or less/more excess water, respectively). In this sense, climate change forces regions from a dry to a drier and from a wet to a wetter regime.

## 4. Conclusions

An attribution analysis of the causes of ecohydrological change was employed to determine (1) the climate-induced external impact in terms of water and energy supply as external forcing, which is distinguished from (2) the internal processes due to anthropogenic influence modifying the partitioning of the surface fluxes compensating the climate-induced forcing [18].

This study applied the ecohydrological model analysis in relation to vegetation greenness change and urbanization characterized by nighttime lighted cities. This diagnostic reveals how regions suffering vegetation greenness reduction show different patterns of energy–water dependence. The results of the attribution analysis of change, which are conditional to vegetation greenness change and urbanization, support a shift from traditional management of resources and risks to an integrated monitoring and holistic response across wide ranges of space–time scales by linking remote sensing, climate, and ecosystem model analysis. The following results are presented as samples for possible management:

(1) Regions where vegetation greenness increased in the second period indicate similar distribution in (*U,W*) space (as for the whole region). That is, a prevailing increase of vegetation growth could be found over the whole of Asia and Australasia since the early 1980s and did not show regional or climate flux dependence. However, regions of decreasing vegetation greenness were concentrated in the water-limited regime, both in the administrative Asia and Australasia regions of significant (*U,W*) change and spatially contiguous nighttime lighted cities.
(2) The attributions of change to external/climate and internal/human-induced effects indicated large-scale afforestation and deforestation occurring mainly in China and Russia, respectively, which is in agreement with previous research about the largest net gain and loss by illegal logging of forest [11,35,36]. Southeast Asia, where a massive conversion of tropical forest to industrial tree plantations [37] occurred, showed few pixels in pink as deforestation (but in light blue as afforestation). The possible cause is that industrial tree plantations there evaporate more than tropical forests, just like forests evaporate more than bare land as observed after afforestation in China (light blue).
(3) Significant changes in (*U,W*) space, which were associated with decreasing vegetation greenness, showed that dry areas remained dry or got dryer, and wet regions remained wet or got wetter, and these changes were separated at the (*D* = 1) threshold line.

In general, complementing geographical presentation of remote sensing information and climate and ecosystem models provides improved understanding of terrestrial biosphere responses to climate and land use change. It will help shift traditional management of resources and risks to integrated monitoring and holistic responses across wide ranges of space–time scales.

## References

[1] Nemani R.R., Keeling C.D., Hashimoto H., Jolly W.M., Piper S.C., Tucker C.J., Myneni R.B., Running S.W. (2003). Climate-Driven Increases in Global Terrestrial Net Primary Production from 1982 to 1999. Science.

[2] Fensholt R., Langanke T., Rasmussen K., Reenberg A., Prince S.D., Tucker C., Scholes R.J., Le Q.B., Bondeau A., Eastman R. (2012). Greenness in Semi-Arid Areas across the Globe 1981–2007—An Earth Observing Satellite Based Analysis of Trends and Drivers. Remote Sens. Environ..

[3] Piao S., Yin G., Tan J., Cheng L., Huang M., Li Y., Liu R., Mao J., Myneni R.B., Peng S. (2015). Detection and Attribution of Vegetation Greening Trend in China over the Last 30 Years. Glob. Chang. Biol..

[4] Xu L., Myneni R.B., Chapin Iii F.S., Callaghan T.V., Pinzon J.E., Tucker C.J., Zhu Z., Bi J., Ciais P., Tømmervik H. (2013). Temperature and Vegetation Seasonality Diminishment over Northern Lands. Nat. Clim. Chang..

[5] Forkel M., Carvalhais N., Rödenbeck C., Keeling R., Heimann M., Thonicke K., Zaehle S., Reichstein M. (2016). Enhanced Seasonal CO_2_ Exchange Caused by Amplified Plant Productivity in Northern Ecosystems. Science.

[6] Keenan T.F., Prentice I.C., Canadell J.G., Williams C.A., Wang H., Raupach M., Collatz G.J. (2016). Recent Pause in the Growth Rate of Atmospheric CO_2_ Due to Enhanced Terrestrial Carbon Uptake. Nat. Commun..

[7] Mao J., Ribes A., Yan B., Shi X., Thornton P.E., Séférian R., Ciais P., Myneni R.B., Douville H., Piao S. (2016). Human-Induced Greening of the Northern Extratropical Land Surface. Nat. Clim. Chang..

[8] Zhu Z., Piao S., Myneni R.B., Huang M., Zeng Z., Canadell J.G., Ciais P., Sitch S., Friedlingstein P., Arneth A. (2016). Greening of the Earth and Its Drivers. Nat. Clim. Chang..

[9] Cheng L., Zhang L., Wang Y.P., Canadell J.G., Chiew F.H., Beringer J., Li L., Miralles D.G., Piao S., Zhang Y. (2017). Recent Increases in Terrestrial Carbon Uptake at Little Cost to the Water Cycle. Nat. Commun..

[10] Bjorkman A.D., Myers-Smith I.H., Elmendorf S.C., Normand S., Rüger N., Beck P.S., Blach-Overgaard A., Blok D., Cornelissen J.H.C., Forbes B.C. (2018). Plant Functional Trait Change across a Warming Tundra Biome. Nature.

[11] Chen C., Park T., Wang X., Piao S., Xu B., Chaturvedi R.K., Fuchs R., Brovkin V., Ciais P., Fensholt R. (2019). China and India Lead in Greening of the World through Land-Use Management. Nat. Sustain..

[12] Liu Y.Y., Van Dijk A.I., De Jeu R.A., Canadell J.G., McCabe M.F., Evans J.P., Wang G. (2015). Recent Reversal in Loss of Global Terrestrial Biomass. Nat. Clim. Chang..

[13] Pan Y., Birdsey R.A., Fang J., Houghton R., Kauppi P.E., Kurz W.A., Phillips O.L., Shvidenko A., Lewis S.L., Canadell J.G. (2011). A Large and Persistent Carbon Sink in the World’s Forests. Science.

[14] Ukkola A.M., Prentice I.C., Keenan T.F., Van Dijk A.I., Viney N.R., Myneni R.B., Bi J. (2016). Reduced Streamflow in Water-Stressed Climates Consistent with CO_2_ Effects on Vegetation. Nat. Clim. Chang..

[15] Cai D., Fraedrich K., Sielmann F., Guan Y., Guo S., Zhang L., Zhu X. (2014). Climate and Vegetation: An ERA-Interim and GIMMS NDVI Analysis. J. Clim..

[16] Renner M., Bernhofer C. (2012). Applying Simple Water-Energy Balance Frameworks to Predict the Climate Sensitivity of Streamflow over the Continental United States. Hydrol. Earth Syst. Sci..

[17] Tomer M.D., Schilling K.E. (2009). A Simple Approach to Distinguish Land-Use and Climate-Change Effects on Watershed Hydrology. J. Hydrol..

[18] Cai D., Fraedrich K., Sielmann F., Guan Y., Guo S. (2016). Land-Cover Characterization and Aridity Changes of South America (1982–2006): An Attribution by Ecohydrological Diagnostics. J. Clim..

[19] Cai D., Fraedrich K., Sielmann F., Zhang L., Zhu X., Guo S., Guan Y. (2015). Vegetation Dynamics on the Tibetan Plateau (1982 to 2006): An Attribution by Eco-Hydrological Diagnostics. J. Clim..

[20] Balsamo G., Albergel C., Beljaars A., Boussetta S., Brun E., Cloke H., Dee D., Dutra E., Muñoz-Sabater J., Pappenberger F. (2015). ERA-Interim/Land: A Global Land Surface Reanalysis Data Set. Hydrol. Earth Syst. Sci..

[21] Tucker C.J., Pinzon J.E., Brown M.E., Slayback D.A., Pak E.W., Mahoney R., Vermote E.F., El Saleous N. (2005). An Extended AVHRR 8-Km NDVI Dataset Compatible with MODIS and SPOT Vegetation NDVI Data. Int. J. Remote Sens..

[22] Elvidge C.D., Baugh K.E., Kihn E.A., Kroehl H.W., Davis E.R. (1997). Mapping City Lights with Nighttime Data from the DMSP Operational Linescan System. Photogramm. Eng. Remote Sens..

[23] Small C., Pozzi F., Elvidge C.D. (2005). Spatial Analysis of Global Urban Extent from DMSP-OLS Night Lights. Remote Sens. Environ..

[24] Small C., Elvidge C.D., Balk D., Montgomery M. (2011). Spatial Scaling of Stable Night Lights. Remote Sens. Environ..

[25] Budyko M.I. (1974). Climate and Life.

[26] Schreiber P. (1904). Über die Beziehungen zwischen dem Niederschlag und der Wasserführung der Flüsse in Mitteleuropa. Z. Meteorol..

[27] Fraedrich K. (2010). A Parsimonious Stochastic Water Reservoir: Schreiber’s 1904 Equation. J. Hydrometeorol..

[28] Milne B.T., Gupta V.K., Restrepo C. (2002). A Scale Invariant Coupling of Plants, Water, Energy, and Terrain. Ecoscience.

[29] Babel W., Biermann T., Coners H., Falge E., Seeber E., Ingrisch J., Schleuß P.M., Gerken T., Leonbacher J., Leipold T. (2014). Pasture Degradation Modifies the Water and Carbon Cycles of the Tibetan Highlands. Biogeosciences.

[30] Gleisner H., Thejll P., Christiansen B., Nielsen J.K. (2015). Recent Global Warming Hiatus Dominated by Low-Latitude Temperature Trends in Surface and Troposphere Data. Geophys. Res. Lett..

[31] Fyfe J.C., Gillett N.P., Zwiers F.W. (2013). Overestimated Global Warming over the Past 20 Years. Nat. Clim. Chang..

[32] Chen B., Zhang X., Tao J., Wu J., Wang J., Shi P., Zhang Y., Yu C. (2014). The Impact of Climate Change and Anthropogenic Activities on Alpine Grassland over the Qinghai-Tibet Plateau. Agric. For. Meteorol..

[33] Fleischer K., Rebel K.T., Van Der Molen M.K., Erisman J.W., Wassen M.J., Van Loon E.E., Montagnani L., Gough C.M., Herbst M., Janssens I.A. (2013). The Contribution of Nitrogen Deposition to the Photosynthetic Capacity of Forests. Glob. Biogeochem. Cycles.

[34] Achard F. (2009). Vital Forest Graphics.

[35] MCPFE (2007). Combating Illegal Harvesting and Related Trade of Forest Products in Europe. Report for the MCPFE Workshop Held in Madrid, Spain, 3–4 November 2005.

[36] Hansen M.C., Stehman S.V., Potapov P.V., Arunarwati B., Stolle F., Pittman K. (2009). Quantifying Changes in the Rates of Forest Clearing in Indonesia from 1990 to 2005 Using Remotely Sensed Data Sets. Environ. Res. Lett..

[37] Röll A., Niu F., Meijide A., Hardanto A., Knohl A., Hölscher D. (2015). Transpiration in an Oil Palm Landscape: Effects of Palm Age. Biogeosciences.

